# Foreign Psychological Literature

**Published:** 1856-01-01

**Authors:** 

**Affiliations:** Medico-Legal Consultation on a Case of Monomania


### FOREIGN PSYCHOLOGICAL LITERATURE.

### *Medico-Legal Consultation on a Case of Monomania.* By M. Baillarger.

The parents of M. M—, who died in the Charenton, 10th Jan. 1851, consulted M. Dubois as to whether deceased was in a sound state of mind at the time of making his will, in June, 1848. The confinement in Charenton dated from Nov. 1850, when he was in a state of dementia and general paralysis. The question to be determined was, whether the mental affection was of recent origin in 1848, or was the last stage of a much older affection existing at the time of the execution of the testament. This was to be answered—1st, by certain writings of deceased; and 2ndly, by the statements of the medical attendants of M. M—.

In July, 1846, M. M— addressed a letter to the "Constitutionnel," giving notice that the police had directions to check the machinations of his enemies. In 1847 he also complained to the *procureur du roi* that he was annoyed by anonymous letters, and by the culpable designs of certain persons to terrify him, and *alterer son intelligence.* A note was also found, written by himself five days before the will was made, and which purported to contain a receipt for an antidote to poison, that had been, it stated, administered to him by a *garde mobile.* The antidote consisted of vanille and milk.

Among the papers belonging to deceased was a list of his enemies written by himself.

The medical attendant of the deceased, Dr. Delente, stated that M. M— had suffered an attack of apoplexy in 1841, the traces of which were visible after death. Dr. Delente had regarded M. M— as a monomaniac, being under the impression that his heirs had conspired to poison him.

From these and other circumstances, M. Dubois gave it as his opinion that the deceased was a monomaniac in 1846; that the mental affection continued; and, that at the date of the execution of the will he was not in a sound state of mind.—"Annales Médico-Psychologiques." Juillet.

---

*On the Identity of Dreaming with Insanity.* By M. Moreau, of Tours.

M. Moreau holds, as a fundamental point in the explanation of the phenomena of insanity, the essential identity of the state of dreaming with insanity. This proposition he regards as the corner-stone of the edifice of the science of psychology. This conviction M. Moreau has arrived at from researches long conducted by himself, and from the study of other writers upon psychology.

The following extracts will illustrate the principal arguments of M. Moreau on this question.

In refuting certain objections taken by M. Delasiauve, M. Moreau observes—

"No dream can be called spontaneous, we do not of our own accord, and according to our own good pleasure, plunge into the state of dreaming; we may prepare all the conditions, we may drive away all the obstacles to sleep, but we cannot dream voluntarily; that is altogether another matter. At the very moment that these conditions for which we have arranged promise the desired result, at that instant we cease to be ourselves; with the inner consciousness our spontaneity of action is lost, the *ego* is transformed; another individuality, that of the dreamer, takes the place of the awake. We see then here that nothing takes place but that which occurs in delirium, in a dream—one is delirious. In the former it is so in obedience to a physiological cause unknown; in the second, in obedience to an unknown pathological cause in spontaneous insanity), or well known (in the madness of intoxication). The result in both cases being the extinction, the slow or sudden annihilation of intellectual spontaneity—a metamorphosis of the *ego*—a dream.

"The state of dreaming has in all ages attracted the attention of physiologists, but has it been sufficiently studied in itself, independently of the organic conditions in which it is produced? Are we not too much accustomed to regard these conditions as indispensable to its development? Has it been ascertained whether it may not be met with in very different pathological and physiological conditions? In other words, must we recognise in the dream a particular manifestation of the thinking faculty, determinable by essentially different causes?

"*a.* We have elsewhere said, that what we call absence of mind, is in reality but incomplete dreaming, an intermediate state between waking and sleeping, and received more with reference to the intellectual than to the properly organic conditions of sleep. We speak indifferently of reverie and absence of mind as an intense mental pre-occupation, which absorbs our attention to things around us. Here, then, common language bears us out in our views.

"It may be said that, in this case, the phenomena of dreaming are produced under normal conditions, the intellect not being vitiated by any morbid influence, which does not in the least reveal the presence of a germ that the slightest cause may suffice to germinate.

"There is a physiological axiom—no sensation, no perception of sensation, without attention. Where then, from any cause whatever, internal impressions absorb the attention, internal impressions are necessarily unheeded, and are as if they were not. The individual is entirely delivered over to his internal reflections, to his meditations; he has broken with the outer world, so far, at least, as regards his thoughts. He is in a veritable dream, always within the limits of the same thoughts.

"*b.* The state of dreaming may be exhibited in its highest degree of development, even where the most important elements of sleep are wanting, for instance, in somnambulists.

"*c.* By the word sleep we understand that particular state which we can only comprehend by, so to say, decomposing and separating the physical from the psychical elements thereof."

Sleep, the author observes, is but a state of suspension, or temporary annihilation, of the thinking faculty, and between this and the state of stupor, whether produced by congestion of the brain or by the poisonous action of opium, hachisch, alcohol, or the inspiration of ether, &c., despite the differences of cause, there is absolute identity of physiological conditions. The objections urged against the identity of dreaming and delirium, M. Moreau attributes to the confusion of two essentially distinct points, the form and the basis (*fond*), the latter remaining uniform, however the former may vary. Apparently, the characters which most differ are, on the one hand, the duration of physiological sleep and its attendant dreams, the great number and variety of objects which constitute the latter; and on the other hand, the intermittence of phenomena of delirium proper, with the restricted limits within which, in ordinary cases, they are confined.

"But these differences are after all (M. Moreau remarks) but differences of form, which, considerable as they may be, do not destroy the identity of the nature of the phenomena themselves.

"So true is this, that one sees these very differences disappear in many acute cases of delirium, in all those caused by alcohol, opiates, &c., and in stupidity, in which the recent researches of M. Baillarger have shown that the play of the imagination is no less capricious, or less extensive, than in ordinary sleep. In many instances, indeed, it would be correct to say that physiological dreams are even more limited and restricted in their conceptions than those to which the name of delirium or insanity is exclusively applied.

"It would be sufficient to infer a greater tenacity and persistence, and the individual would be then positively and absolutely mad.

"This supposition, moreover, is borne out by more facts than generally supposed; thus there are many insane persons who trace their delirious ideas, or convictions, or hallucinations, to a dream. With many, insanity is in reality but the continuation of the dream.

"In confirmed insanity the dreams have effected such profound impressions upon the organism, that they cannot be effaced by the waking condition."

The impressions of dreams are sometimes so vivid that it is difficult to divest oneself of the idea of their reality.

"*This is certainly a moment of insanity* (observes M. Moreau, who adds):—In order that the insanity shall continue, we have only to imagine that the fibres of the brain have suffered too violent a shock to have recovered themselves. The same thing may occur more slowly."

M. Moreau supports his own views by quotations from Condillac, Sauvages, Virey, Spinoza, Van Helmont, and other writers.

"Insanity (the author proceeds to observe) implies a real transformation of personality. There are cases in which this transformation is so evident that the line of demarcation between the wakeful and the dreaming states could not be more clear and precise. There are among the insane, for instance, persons whose whole life previously to the delirium offers not a trace of its existence.

"Every act of the thinking faculty, performed without our free and voluntary assent, appertains to the state of dreaming.

"The mind cannot quit its ordinary wakeful condition without passing into that of dreaming; in any other state its actions must be destroyed or suspended, as in profound sleep or coma.

"Sleep may be compared to the repose of a pendulum, which is still susceptible of new oscillations under the slightest impulse, so long as the machinery by which it is set in motion retains its integrity. Death may be closely represented by this same pendulum, when the destruction of its wheels has put it for ever beyond the possibility of moving. Time exists only in relation to the succession of our thoughts.

"It follows from what has been said, that the impression made upon the thinking faculty by loss of consciousness is the same, whatever may be its duration. Every person can recal that of which he is sensible at the moment of waking; whatever may be the length of time that has lapsed during sleep, the state of the mind is the same; there is a feeling of a new existence, the primary elements of which are supplied by the memory. It is perfectly true that in this state there is no difference between a moment and an age, so that if an individual were to wake at the end of many thousand years, his first impressions would not differ from what he would have experienced had he slept only a few hours.

"What occurs when an individual who has been transformed by delirium, and coming to himself with his inner consciousness annihilated, recovers his reason after ten, fifteen, or thirty years of mental disease? Exactly what would have occurred had he woke from several hours' sleep. He is surprised that he does not find everything in the same state as at the moment that he was struck by insanity. His eyes seek the old objects, his affections look for the same persons; every person and object around him, and among which he has passed so many years, he sees now for the first time, or has but a confused recollection of having seen them. He cannot recognise in the grown-up persons around him his own children; he is not sure of his own identity,—whence these wrinkles, these grey hairs, these indications of advanced age?"

M. Moreau concludes his paper in the following *resumé*:—

"It is not easy to allow that a body can be propelled simultaneously in two opposite directions.

"Morally, likewise, whether it affirm or deny, the mind is in either the affirmation or negation entirely one and the same, successively or alternately—but never simultaneously.

"Now, that which the delirious individual denies, he would in the normal state have affirmed. Moreover, since delirium, as we have described it, does not affect the essence of the intellectual powers, or if one may so speak, the internal economy of those faculties, but with an uniform relation to certain objects on which their action is directed, these faculties remain intact, even amidst their false and extravagant perceptions; so that we may say, without transgressing psychological truth, that the insane are such as they are according to the point of view from which they are regarded. It follows that not to admit the absolute transformation of the *ego* in the state of delirium, is to declare that the soul may deny without ceasing to affirm, or in other words, may simultaneously undergo two modifications — two modes of existence which destroy one another."

As naturally connected with the preceding notice of M. Moreau's paper in the "Annales Psychologiques," we append an abstract of the Report of the Commission of the Imperial Academy of Medicine, by M. Bousquet, May 1855, upon a memoir of M. Moreau, on the pathological anatomy of delirium; and a brief notice of the discussion thereon.

The Report takes exception to the views of M. Moreau, who, it observes, regards insanity, like other diseases, as an organic disease. The reporter charges M. Moreau with having confusedly used the words delirium and insanity, not regarding the distinction between the two: the one being a temporary condition, they remark, the other more durable, often lasting for life, and transmitted hereditarily; the one generally combined with fever and constitutional disturbance, the other compatible with perfect bodily health. M. Moreau, remarks M. Bousquet, considers that insanity has been viewed too much as independent of organization, and the dominant idea of his memoir is to restore it to its proper place. The reporter, as opposed to M. Moreau, holds that that source of the mental disorder is to be sought sometimes in the brain, sometimes elsewhere. The exclusive theory of either one or the other opinion they look upon as erroneous. The most opposite condition of the nervous centres may influence and implicate the brain; in these cases, insanity may be an eventual contingent effect. Reasoning from physiological analogy, it is urged that as impressions are conveyed from the periphery to the nervous centres, and *vice versâ*, so a similar relation of causation may occur in insanity. Besides which, those who attribute mental disease in all cases to structural change in the brain, contravene their own fundamental doctrine, which is not to admit anything of which they have not the evidence of their senses. The reporter further urges the difference between the temporary condition of the brain during sleep, and the permanent state of insanity transmissible hereditarily. The views of M. Moreau they consider to be the misapprehension of resemblance for identity.

M. Baillarger criticised and opposed the Report, which, he observed, contained only negative propositions; the general conclusion of which was to the effect, that as we are not able to unravel the mysteries of the subject, we should therefore expose all our doubts and conceal our hopes. The study of mental diseases, observed M. Baillarger, would not present the attractions it does, or be followed with so much ardour, if it were as fruitless as indicated in the Report. M. Baillarger defended M. Moreau, on the ground that his object was not to affirm the exclusively material nature of insanity, but to endeavour to counteract extreme spiritualistic notions, which to a too great extent influence practice. M. Baillarger further urged in defence of M. Moreau's opinions, that the author had enforced only a strong analogy between insanity and sleep.

M. Londe drew a distinction between delirium and insanity; in the former, he recognised only a disorder of the intellect; in the latter, other functions also are deranged. M. Londe objected to the strictly physiological view of the opinion that insanity cannot exist without organic disease. M. Londe further criticised the objection of M. Bousquet, that if insanity be analogous with dreaming, then do we lose our reason nightly, and recover it in the morning. This view, said M. Londe, is too spiritual, for that natural sleep does not overtake us without our having some idea of its approach; but if before sleeping the brain be excited by drinking, or by emotions, or by work, it is attended with dreams more or less fatiguing. With reference to the failure of pathologists to detect any certain alteration of the brain in insanity, M. Londe observed that similar failure had occurred in other diseases. M. Londe, in concluding, stated his opinion to be that, in recent and curable cases of insanity, the causes, chagrin, &c., have acted upon only a portion of the brain; while in more serious forms of the affection—*e. g.*, following violent disturbance, febrile delirium, &c.—the lesion has been general, or has become so, whence not only the intellect, but also other cerebral functions, are injured.

M. Ferrus opposed the Report, which he characterized as having for its object to exhume and diffuse exclusive doctrines.

M. Piorry combated the arguments of M. Bousquet from the difference in the permanency of insanity as compared with that of delirium. This, observed M. Piorry, cannot be regarded as a ground of distinction, since cases of delirium with fever are met with lasting ten, fifteen, twenty days, or even longer; while there are cases of delirium without fever which should be regarded as insanity, passing off within a week: the absence of fever, according to M. Bousquet, distinguishes insanity from delirium. Insanity, again, does not always develop itself slowly, but often breaks out as suddenly as delirium.

In answer to the objection urged by M. Bousquet against the analogy drawn by M. Moreau between insanity and dreaming, M. Piorry observed, that it is by such physiological studies that psychological science is to be advanced.

With reference to the statements of M. Bousquet that morbid anatomy has thrown but little light upon the nature and cause of insanity, M. Piorry urged that it does not follow, that because in so delicate and complex an organ as the brain many instances occur in which nothing of value has been found, therefore that those numerous instances should be lost sight of in which sufficient has been found to account for the perversion of the thinking faculties.

The Report of the discussion upon this, M. Bousquet's paper, occupies eighty-two pages. We cannot afford space for its reproduction. It may suffice to say that it was severely criticised, and its principal positions refuted by various speakers.

### *Pathological Condition of the Brain in Epileptics.*

At the *Societé Medico-psychologique*, January 19, 1855, M. Delasiauve observed how frequently no appreciable alteration of structure can be detected after death in epileptics; while on the other hand, the most varied *post-mortem* appearances are sometimes met with, in which the etiological relationship is not traceable. He had recently met with a case in which a large osseous plate, from three to four centimetres in extent (=1.181 to 1.574 English inches), was placed in the form of an arc between the two hemispheres of the brain, and appeared as if a portion of the falx were ossified. Was this growth, asked M. Delasiauve, the cause of the convulsions? It would not seem from its position, that it could have much interfered with the functions of the brain. The symptoms, moreover, seem to have coincided with an apoplectic seizure dating about four years previously, and which had left a large cell in the substance of the middle lobe of the left hemisphere, as evidence of its occurrence. There had been three or four paroxysms at monthly intervals; during the last few months the dementia and general paralysis had made great progress.

Another patient had presented a more striking ossific deposit. This was a fragment of bone, of a cubic form, about the size of a filbert, and studded all over with needle-shaped spiculæ. This fragment was lodged in the fissure of Sylvius, adhering slightly to the pia mater. It is probable that these spiculæ irritated the otherwise healthy brain and gave rise to the attacks. The patient had frequently suffered from vomiting, both at the time of the fits, and in the intervals. It was not clear whether these attacks of vomiting were referrible to the cerebral irritation, or to onanism, to which the patient had abandoned himself.

M. Loiseau related the case of an epileptic in whom it was found that an osseous growth had caused absorption of a portion of the hemisphere.

M. Ferrus related the case of a lady of high intellectual development, who had suffered from intense pain in the anterior region of the brain, and on one occasion an epileptic seizure occurred. It was found that an osseous tuft had been formed on the orbitar plate, which had torn the membranes and injured the brain.

---

### *A Peculiar Form of Insanity in Children.*

M. Delasiauve brought this subject under the notice of the *Société Médico-psychologique*, February 26th. This affection M. Delasiauve described as having for its fundamental character a disturbance of the intellectual faculties, manifested more or less confusion of ideas, but was always complicated with ecstatic phenomena, the paroxysms of which varied in duration, and in some cases returned at short intervals. The patients remained several hours of the day as if wrapt in a sort of mystical contemplation. Often the attention was fixedly directed to one spot from which not even the most vivid impressions could arouse them. In other cases the attention was alternately directed to different points. The limbs and body were placed in the most grotesque attitudes and positions; sometimes the head was bent in forced directions, sometimes the arms and legs remained elevated and extended. In some of these cases there was seen slow and measured jactitation, after the fashion of Punchinello. Of the eight or nine cases seen by M. Delasiauve almost all were cured within a limited period, in some cases with relapse. Bathing, sulphate of quinine, and attention to hygiene, were followed by successful results.

Although these cases were important, they did not, in the opinion of M. Delasiauve, deserve a special nomenclature as a new form of mental disease. The phenomena of these cases do not belong to mania, nor specially to early age. They are met with in those forms of partial insanity attended with convulsions, such as catalepsy and epilepsy. The ecstatic state corresponds to a slight degree of cerebral erethism, whereby the intellect, acting through volition, is subordinated to the automatic organic system. Hence, if this view be correct, ecstasy may take place whenever from moral or physical causes the normal activity of the nervous centres is increased, and favours the production of spasm. The preference of these attacks shown towards early age may be explained by the greater impressionability of that time of life. In some instances this special predisposition is referable to onanism, or intimidation, which either depress or concentrate the nervous sensibility. Several of the patients were addicted to the solitary vice, nearly all had been the subject of cruelty or unjust rigour, or had been frightened by exaggerated representations of their offences, and by fear of the wrath of God.

M. Moreau had met with instances of this form of affection, and regarded them as cases of epilepsy, attended with a degree of stupor, offering some resemblance to ecstasy.

M. Belhomme inquired if M. Delasiauve considered that an analogy existed between ecstasy and catalepsy?

M. Delasiauve in reply, stated that he recognised in ecstasy a state of muscular immobility without contraction, accompanying a particular cerebral disorder, while in catalepsy there was abolition of feeling with tetanic rigidity. The difference is one rather of degree than of kind. Ecstasy seems to be a slighter degree of this cataleptic state.

M. Alfred Maury, mentioned an epidemic melancholy which had prevailed among the inhabitants of a district in Siberia, some years ago, under the influence of a Buddhist prediction. In this disorder the sufferers uttered a sad monotonous chant, concluding with a paroxysm of excitement, which was followed by insanity or restoration. The moral and physical condition of this people resembled that of childhood.

M. Buchez did not consider that either of the speakers had elucidated the phenomena related by M. Delasiauve. He would ask whether ecstasy occurring in the insane and epileptic is of the same nature as ecstasy occurring in the previously healthy and in those persons who can induce the ecstatic condition by profound meditation? He further asked, whether ecstasy and catalepsy are physiologically the same? whether they might exist separately? whether they have the same organic seal?

M. Baillarger had seen cases of melancholy stupor pass into ecstasy. He objected to the use of the word "physiological" to express conditions referred to in this discussion, and which he considered as strictly pathological. For instance, if ecstasy be a suspension of the intellectual powers, how can it be said to be a physiological state?

M. Alfred Maury compared the state of ecstasy to the passing delirium of fever, which, frequently recurring, may pass into insanity.

M. Buchez considered that there was a great analogy, but not an identity, between the ecstasy occurring in health and that occurring in disease. M. Buchez further illustrated his opinions by reference to the state of internal abstraction or contemplation under which musical composers, without the presence of a musical instrument, veritably hear the pieces they compose; and a painter sees in imagination the persons he transfers to his canvas.

M. Ferrus asked if a person in a state of ecstasy was, medico-legally speaking, responsible for his acts; M. Ferrus promised to lay before the Society the particulars of some cases bearing upon this point.

---

*On the Organic Cause of Mental Alienation, accompanied by General Paralysis.* By M. Bayle, who lays down the following as the chief points of his Paper:—

1. There is a particular species of mental affection, of a symptomatic character, perfectly distinct from the essential forms of alienation, and forming a malady by itself, an individual malady, having its own causes, with symptoms and anatomical characters too distinct to permit of their being confounded with any other affection.

2. Its causes have one common effect in producing slow or sudden congestion of the vessels of the pia mater and brain.

3. The symptoms may be reduced to two, which commence and progress concurrently—viz., paralysis general and incomplete, and non-febrile delirium with great feebleness of the faculties. The paralysis makes constant progress in the course of disease, and terminates in almost entire privation of voluntary movements. The delirium has the peculiar feature of being characterized by ambition, and passing successively through the forms of monomania, mania, and dementia. Frequently, mania is wanting.

4. The anatomical characters are those of chronic inflammation of the membranes of the convexity of the cerebral hemispheres, often extending to the subjacent surface of the substance of the brain itself.

The proofs of these positions are deduced from the post-mortem examination of the brains of insane paralytics compared with the brains of sane individuals, and from the analogy of this disease with other inflammations of serous membranes.

Among the lesions discovered in the examination of one hundred bodies, and which were characteristic of chronic inflammation of the membranes of the brain, some were met with in all cases, while others were absent in a certain number. The changes constantly met with were opacity, thickening, increased toughness of the arachnoid to such an extent that sometimes it was possible to suspend a slice of brain by its means without tearing it; extreme congestion of the pia mater; thickening of the arachnoid of the ventricles, which also was covered with granulations; considerable effusion of serum into the cavities of the ventricles and into the network of vessels of the pia mater.

The morbid appearances less frequently met with were adhesions of the membrane, and the softening of the surface of the convolutions; false membranes, or extravasated blood. The substance of the brain was softer in a few cases; in some it was firmer; in the majority it retained its natural consistence.

These post-mortem appearances are not met with in other diseases than chronic meningitis; the slight opacities, &c., occurring towards the close of life in other forms of cerebral disease, do not offer even an analogy. They have always been found by M. Bayle after death from general paralysis, and never in the case of patients who have died from other maladies; hence it is inferred that chronic meningitis is the organic cause of insanity with general paralysis.

---

*Structure of the Cortical Layer of the Convolutions of the Brain.*

M. Baillarger refers to his previous discovery of six alternate layers of grey and white matter in the cortical layer of the brain, and states that MM. Foville and Gratiolet, in confirming his results, have added the existence of another layer of white matter. This last layer follows internally all the folds of the cortical layer, as the pia mater follows them externally. The cortical layer of the convolutions separates itself distinctly from the white substance, especially at the depths of the anfractuosities; it remains, in effect, as if doubled by a very thin layer of this substance. This is a fact which, observes M. Baillarger, he had himself more than once observed, but to which he had not attached the importance it merits. M. Baillarger does not, in the present communication, examine the structure of this white layer, nor the nature of its connexion with the grey substance and the radiating fibres. The present notice records a fact in pathological anatomy, the separation of a group of convolutions from the white substance, and lined by this seventh lamina, such as he has often met with in the sheep.

A female, thirty-seven years of age, was admitted into the Salpêtrière, suffering from symptoms of general paralysis; at the end of eighteen months she died. The left hemisphere of the brain was found softened throughout its whole extent. The attempt to raise the thickened membrane brought with it the entire cortical layer and a portion of the white substance of the brain. At the anterior and superior part it was possible thus to raise and separate an entire group of convolutions. On examining these convolutions in their then reversed position, their summits were smooth and of a bluish-white colour. On cutting into the summit it was observed that the white layer which covered the cortical substance was grey and very thin, of a uniform consistence, and tolerably adherent. This white lamina is the layer described by MM. Foville and Gratiolet as the seventh layer of the cortical substance of the brain.—"Annales Médico-Psychologiques." Janvier.

---

"The American Journal of Insanity," July 1855, contains a history of the several attacks of insanity under which his Majesty King

George the Third suffered. These particulars, which have not hitherto been published in any collected form in this country, have been collected from "parliamentary reports, public papers, political squibs, diaries of persons about the court, tittle-tattle sent to other nations," &c., and have now been embodied in the form of a narrative by Dr. Ray, of Butler Hospital, Providence, United States. We take leave, however, to question whether the result is worthy of all the labour it must have cost. We were in England too well aware of the melancholy fact of the King's insanity at the time, if not familiar with all the details. We have had too much reason to regret the consequences thereof to wish to revive so painful a subject. We are very well convinced that much indirect good has also been providentially educed from that national affliction, by the countenance it gave to improvement in the general principle of the treatment of the insane. Nevertheless, so much being taken for granted, we fail to perceive the benefit that can accrue to psychological medicine from a revival of scenes so painful, and from a history which, after all the laborious research of Dr. Ray, is still imperfect.

Our transatlantic brethren may probably trace in the results of the mental disorder of George the Third, their own elevation to a national status. We may be disposed to concur in the inference, but content to submit with the best grace we may to the dispensations of Providence, we would not raise the veil that screens the domestic griefs of royalty—in the belief that a sovereign has as inalienable a right to have his home held sacred as has the meanest citizen of a republic. And notwithstanding we may incur the imputation of a squeamish tenderness, we hold that such a narrative as the history of the insanity of the King goes very near to trench upon the inviolability of professional confidence. In the ordinary publication of cases by medical men, names are usually suppressed—and where for the ends of justice both names and particulars must be made known, still no more than is required for the furtherance of judicial objects is usually laid bare to public gaze. Why, then, should not the same measure be meted out to the most exalted member of society?

